# Supplementary material for: Systematic Pan-Cancer Analysis Reveals X-C Motif Chemokine Receptor 1 as a Prognostic and Immunological Biomarker
Source: Genes (Basel). 2023 Oct 19;14(10):1961. doi: 10.3390/genes14101961 (PMC10606244; doi:10.3390/genes14101961)
Supplement: Supplementary file 1 [file genes-14-01961-s001.zip › genes-2643436-supplementary.pdf]

Supplementary data for

Systematic Pan-Cancer Analysis Reveals X-C Motif Chemokine Receptor 1as a Prognostic and Immunological Biomarker

Likun Cui [1,†], Liye Zhu [1,†], Jie Chen [1], Chunzhen Li [1], Yizhi Yu [1,*] and Sheng Xu [1,2,*]

[1] National Key Laboratory of Medical Immunology and Institute of Immunology, Naval Medical University, Shanghai 200433, China; poppy199609@163.com (L.C.); zhuly339@163.com (L.Z.); chenj12112021@163.com (J.C.); chunzhenli@smmu.edu.cn (C.L.)

[2] Shanghai Institute of Stem Cell Research and Clinical Translation, Shanghai 200120, China

* Correspondence: yuyz@immunol.org (Y.Y.); xusheng@immunol.org (S.X.)

† These authors contributed equally to this work.

Supplementary Table S1. Primers of *XCR1* for quantitative real-time PCR.

Primer	Sequence
XCR1-F	ATGGAGTCCTCAGGCAACC
XCR1-R	CGAGGGTAGCAAAGACCCA
GAPDH-F	CTGGGCTACACTGAGCACC
GAPDH-R	AAGTGGTCGTTGAGGGCAATG
CXCL9-F	CCAGTAGTGAGAAAGGGTCGC
CXCL9-R	AGGGCTTGGGGCAAATTGTT
CXCR3-F	TTTGACCGCTACCTGAACATAGT
CXCR3-R	GGGAAGTTGTATTGGCAGTGG

Supplementary Table S2. Cox regression analysis in LIHC *.

Characteristic	Low expression of *XCR1*	High expression of *XCR1*	p
n	187	187	
T stage, n (%)			0.009
T1	77 (20.8%)	106 (28.6%)	
T2	52 (14%)	43 (11.6%)	
T3	51 (13.7%)	29 (7.8%)	
T4	7 (1.9%)	6 (1.6%)	
N stage, n (%)			1.000
N0	133 (51.6%)	121 (46.9%)	
N1	2 (0.8%)	2 (0.8%)	
M stage, n (%)			0.626
M0	142 (52.2%)	126 (46.3%)	
M1	3 (1.1%)	1 (0.4%)	
Pathologic stage, n (%)			0.024
Stage I	73 (20.9%)	100 (28.6%)	
Stage II	46 (13.1%)	41 (11.7%)	
Stage III	52 (14.9%)	33 (9.4%)	

Characteristic	Low expression of *XCR1*	High expression of *XCR1*	p
Stage IV	3 (0.9%)	2 (0.6%)	
Tumor status, n (%)			0.005
Tumor free	86 (24.2%)	116 (32.7%)	
With tumor	89 (25.1%)	64 (18%)	
Gender, n (%)			0.269
Female	55 (14.7%)	66 (17.6%)	
Male	132 (35.3%)	121 (32.4%)	
BMI, n (%)			0.207
<=25	94 (27.9%)	83 (24.6%)	
>25	73 (21.7%)	87 (25.8%)	
Age, n (%)			0.196
<=60	82 (22%)	95 (25.5%)	
>60	105 (28.2%)	91 (24.4%)	
OS event, n (%)			< 0.001
Alive	104 (27.8%)	140 (37.4%)	
Dead	83 (22.2%)	47 (12.6%)	
DSS event, n (%)			0.002
Alive	129 (35.2%)	158 (43.2%)	
Dead	52 (14.2%)	27 (7.4%)	
PFI event, n (%)			0.004
Alive	81 (21.7%)	110 (29.4%)	
Dead	106 (28.3%)	77 (20.6%)	
Histologic grade, n (%)			0.712
G1	27 (7.3%)	28 (7.6%)	
G2	88 (23.8%)	90 (24.4%)	

Characteristic	Low expression of *XCR1*	High expression of *XCR1*	p
G3	62 (16.8%)	62 (16.8%)	
G4	8 (2.2%)	4 (1.1%)	
Residual tumor, n (%)			0.621
R0	165 (47.8%)	162 (47%)	
R1	10 (2.9%)	7 (2%)	
R2	1 (0.3%)	0 (0%)	
Adjacent hepatic tissue inflammation, n (%)			0.604
None	57 (24.1%)	61 (25.7%)	
Mild	52 (21.9%)	49 (20.7%)	
Severe	7 (3%)	11 (4.6%)	
Vascular invasion, n (%)			0.360
No	97 (30.5%)	111 (34.9%)	
Yes	58 (18.2%)	52 (16.4%)	

* Data was from RNAseq data in the level 3 HTSeq-FPKM format of the TCGA (https://portal.gdc.cancer.gov/) LIHC project.

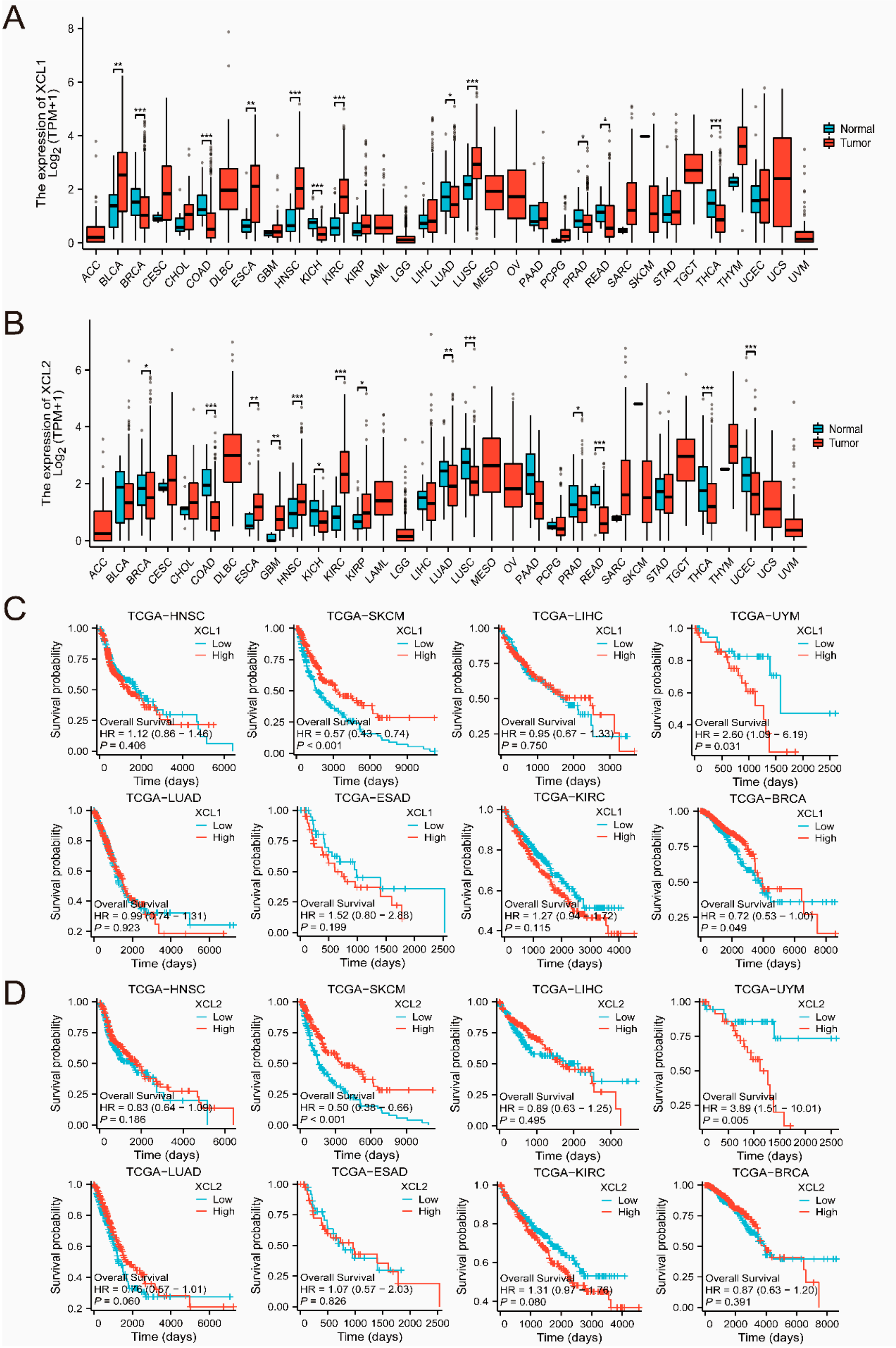

Supplementary Figure S1. Expression of *XCL1/2* and Overall Survival (OS) analysis in pan-caners based on TCGA database

(A-B) Human *XCL1* and *XCL2* expression levels in pan-cancer with its para-cancerous normal tissues from TCGA database. (C-D) Overall Survival analysis of *XCL1* and *XCL2* among several types of tumors in the TCGA database.

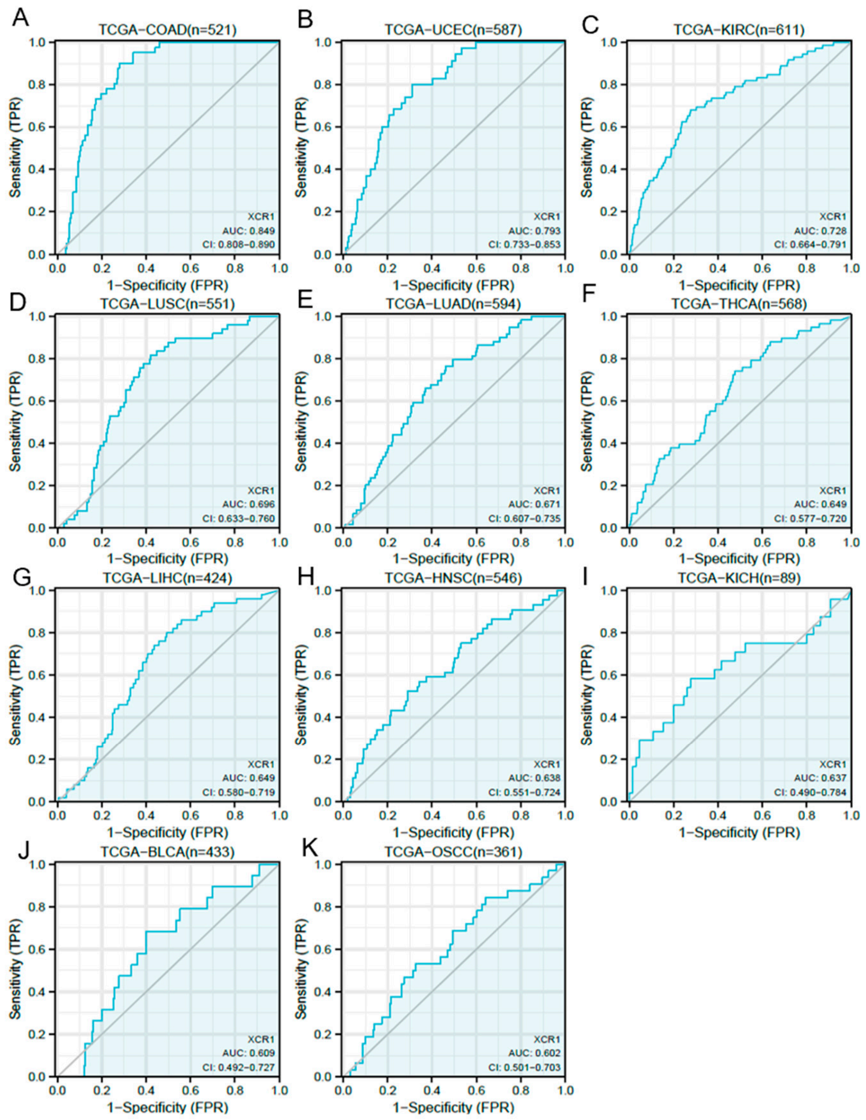

Supplementary Figure S2. Receiver operating curves showing relationship of sensitivity and 1 specificity based on *XCR1* expression in various cancers

(A-K) AUC: area under Curve; CI: confidence interval, Sensitivity/True Positive Rate (TPR): the number of true positive samples detected divides by the number of all true positive samples; Specificity: the number of true negative samples detected divides by the number of all true negative samples; 1- Specificity: the number of false-positive samples detected divides by the number of all true negative samples.

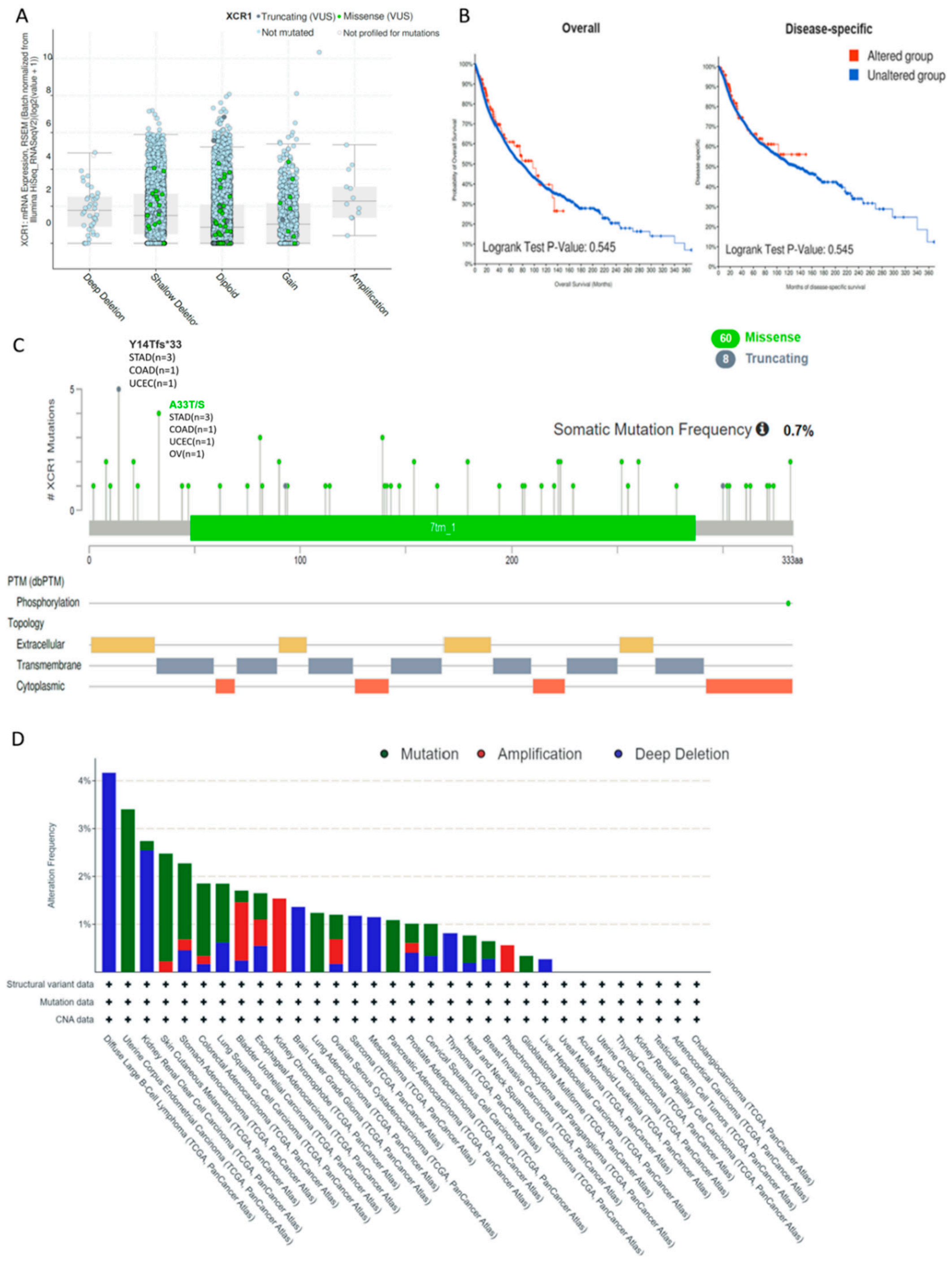

Supplementary Figure S3. Mutation features of *XCR1* in pan-cancer

(A) Association of *XCR1* copy number alteration with its mRNA expression in the TCGA cancer cohort. (B)Association between genetic alteration of *XCR1* and clinical survival (OS/DSS). (C) The alteration frequency with mutation type and mutation site. (D) Alteration frequency with the mutation types of *XCR1* in human pan-cancer.

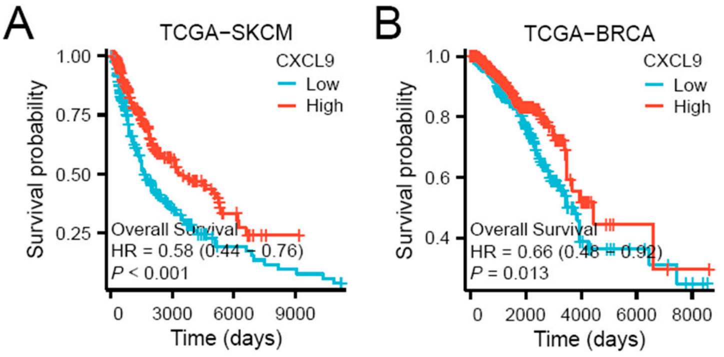

Supplementary Figure S4. Survival prognostic analysis of the expression of *CXCL9*

(A-C) Overall Survival analysis of *CXCL9* among SKCM, BRCA, and LIHC.
